# Regulating the Physicochemical Properties of Chitosan Films through Concentration and Neutralization

**DOI:** 10.3390/foods11111657

**Published:** 2022-06-05

**Authors:** Jie Xu, Kun Liu, Wei Chang, Bor-Sen Chiou, Maoshen Chen, Fei Liu

**Affiliations:** 1 State Key Laboratory of Food Science and Technology, Jiangnan University, Wuxi 214122, China; 17865561788@163.com (J.X.); aqliukun@163.com (K.L.); fliua15c@163.com (W.C.); chenmaoshen@jiangnan.edu.cn (M.C.); 2 Science Center for Future Foods, Jiangnan University, Wuxi 214122, China; 3 School of Food Science and Technology, Jiangnan University, Wuxi 214122, China; 4 International Joint Laboratory on Food Safety, Jiangnan University, Wuxi 214122, China; 5 Western Regional Research Center, ARS, U.S. Department of Agriculture, Albany, CA 94710, USA; bor-sen.chiou@usda.gov

**Keywords:** chitosan films, physicochemical properties, concentration, neutralization

## Abstract

Chitosan offers real potential for use in food preservation, biomedicine, and environmental applications due to its excellent functional properties, such as ease in the fabrication of large films, biocompatibility, and antibacterial properties. However, the production and application of chitosan films were limited by their strong residual acetic acid taste, weak mechanical properties, and poor water vapor barrier properties. In this study, the effects of the chitosan concentration in the film-forming solutions and the neutralization treatment on the physicochemical properties of chitosan films were examined. The results demonstrated that the chitosan concentration affected the mechanical and barrier properties of chitosan films without the neutralization treatment. This was mainly due to the low acetic acid contents in chitosan films after drying. Acetic acid acted as a plasticizer within chitosan films resulting in a looser network structure. After neutralization, the chitosan films showed improvements in properties, with little effect on the chitosan concentration in the film-forming solutions. Moreover, chitosan films after neutralization showed no residual acetic acid. Therefore, neutralization could effectively improve the performance of chitosan films.

## 1. Introduction

In the modern food industry, food packaging is an ubiquitous and important part of food transportation and storage [1]. At present, most of the packaging materials used in the food industry are derived from the petrochemical industry, such as polyvinylidene chloride (PVDC), polyvinyl chloride (PVC), and other plastic films. These plastics cannot be degraded by natural processes; therefore they will have a substantial impact on the environment [2]. Recently, environmentally friendly materials based on polysaccharides, lipids, and proteins have been developed for packaging applications.

Chitosan is mainly derived from shrimp shells, crab shells, microbes, and insects and comprises the second largest natural polysaccharide source after cellulose [3]. In addition, it has been widely used in food preservation [4], biomedicine [5], and environmental [6] applications due to its excellent functional properties, such as ease in the fabrication of large films, biocompatibility, and antibacterial properties. However, films based on chitosan have many drawbacks, including strong residual acetic acid taste, weak mechanical properties, and poor water vapor barrier properties, which limit their applications. Previous studies have reported that the concentration of film-forming solutions and neutralization conditions could affect the mechanical and barrier properties of chitosan films [7,8]. Therefore, the optimization of the preparation conditions can improve the mechanical and barrier properties of chitosan films.

The chitosan concentration in the film-forming solutions determines the number of chitosan molecules per unit volume. Higher chitosan concentrations result in strong interactions between chitosan molecular chains and an increase in intermolecular crosslinking. This leads to a relatively compact film structure. Moreover, higher chitosan concentrations can lead to a more viscous film-forming solution, incomplete degassing, and the formation of bubbles during the film-forming process. This has adverse effects on the mechanical and barrier properties of chitosan films [9]. However, lower chitosan concentrations lead to a relatively loose chitosan film structure. Therefore, it is necessary to select the appropriate chitosan concentration in the film-forming solution.

Chitosan is insoluble in water and requires the use of acetic acid as a solvent. However, the residual acetic acid in the chitosan films will lead to a sour taste, change the density of the film, and affect the film’s crystal configuration, resulting in worse mechanical and barrier properties [10]. Neutralization is a common method to improve the properties of chitosan films. According to He et al., the water resistance of chitosan films can be improved by neutralization. Meanwhile, the mechanical properties of the chitosan films are affected by the types of neutralization solutions, such as sodium hydroxide (NaOH) aqueous solution, alcohol solution or alcohol mixed solution containing NaOH [11].

There are relatively few reports on the effects of chitosan concentration in film-forming solutions and acetic acid contents on the properties of chitosan films. In this study, we examined the effects of chitosan concentration in the film-forming solutions and neutralization treatment on the mechanical and barrier properties of the films. The chitosan films were prepared by the casting method and the mechanism of the effects of chitosan concentration and neutralization on film properties was analyzed, especially the mechanical properties of chitosan films and their relationship with the crystallinity of the materials used in the formation process.

## 2. Materials and Methods

### 2.1. Materials and Reagents

Chitosan (CS, Mw = 500 kDa, degree of deacetylation of 85~90%) was obtained from Pharmaceutical Chemical Reagent Co., Ltd. (Shanghai, China). Sodium hydroxide, hydrochloric acid, boric acid, acetic acid, and glycerin were obtained from Sinopharm Chemical Reagent Co., Ltd. (Shanghai, China). Sodium dihydrogen and phosphate acid were obtained from Maclean Chemical Reagent Co., Ltd. (Shanghai, China).

### 2.2. Preparation of Chitosan Films with Different Film-Forming Solution Concentrations

As a first step, pure acetic acid was added to deionized water to prepare 1.0% (*w*/*v*) acetic acid solution. Then, chitosan powder was added to the acetic acid solution to obtain 0.5, 1.0, 1.5, and 2.0% (*w*/*v*) chitosan solutions and stirred at room temperature for 2.5 h. Thereafter, 10% (*w*/*w*) glycerin was added to the solutions as a plasticizer. The films were cast by pouring 80.0, 40.0, 26.7, and 20.0 mL of the 0.5, 1.0, 1.5, and 2.0% (*w*/*v*) chitosan solutions, respectively, into a square plastic petri dish (10 × 10 cm). The films were dried at 45 °C for 12 h. Following this step, the dried chitosan films were placed in an acrylic drying cabinet and stored at 25 ± 1 °C and 52% ± 1% relative humidity for at least 72 h, which are unneutralized films.

Part of the films that were stored for 24 h in the acrylic dryer were then neutralized with 5% (*w*/*w*) sodium hydroxide solution, washed fully with 1 L deionized water for 5 min to remove excess acid and alkali, and obtain the neutral condition. Finally, the films were dried at 45 °C for 12 h and stored for at least 72 h in an acrylic drying cabinet at 25 ± 1 °C and 52% ± 1% relative humidity before each test.

### 2.3. Mechanical Properties

In accordance with the ASTM standard method D882 [12], the mechanical properties (tensile strength (TS) and elongation at break (EB)) of chitosan films were measured by texture analyzer (TA-XT plus, Stable Micro Systems, Surrey, UK) with A/TG probe. The chitosan film was cut into strips (2 × 8 cm) and the average thickness was determined at 5 random points using a spiral micrometer. The original upper and lower clamping distance, stretching speed, and stretching length of texture analyzer were 50 mm, 0.5 mm/s, and 10 mm, respectively. The force curve of deformation during the tensile process was recorded by Texture Expert Exceed software (Stable Micro Systems, Surrey, UK). The tensile strength (TS) was calculated using:

(1) $$\mathrm{TS}\;=\frac{F}{L\times W},\;$$

where *F* is the axial tensile force (N), *L* is the film thickness (mm), and *W* is the film width (mm). The elongation at break (EB) was determined as follows:

(2) $$\mathrm{EB}\left(\%\right)=\frac{L_{1}-L_{0}}{L_{0}}\times 100,\;$$

where *L*_0_ is the length of sample before stretching (mm) and *L*_1_ is length of sample after stretching (mm).

### 2.4. Water Vapor Permeability

In accordance with the ASTM method E96/E96M [13], the water vapor permeability (WVP) and water vapor transmission rate (WVTR) of chitosan films were measured by the weighing method. Then, 10 mL of deionized water was added to the 2 × 5 cm cup and a chitosan film, which was cut into square pieces (4 × 4 cm), covered, and sealed with parafilm. The original mass of the cup was measured and placed in a desiccator filled with anhydrous silica gel. The final mass of the cup was measured after 72 h. The WVTR of the film was determined in accordance with Liu et al. [14]:

(3) $$\mathrm{WVTR}=\frac{m_{3}-m_{2}}{5.728\times 10^{-4}\times 72},\;$$

where *m*_2_ is the original mass (g), *m*_3_ is the final mass (g), and 5.728 × 10^−4^ is the effective area of the film covering the cup (*m*_2_). The WVP of the film was determined using:

(4) $$\mathrm{WVP}=\frac{WVTP\times L_{a}}{{PA}_{1\;}{-\;PA}_{2}},\;$$

where *L_a_* is the average thickness of the film (mm), *PA*_1_ is the air pressure on the outer surface of the chitosan film (Pa), and *PA*_2_ is the air partial pressure on the inner surface of the chitosan film (Pa).

### 2.5. Water Contact Angle

The water contact angle of the chitosan films was measured using the optical video contact angle meter (OCA 15EC, Dataphysics, Filderstadt, Germany) and the sessile drop method used by Kraisit et al. [15]. A 1 mL flat-head needle syringe was used to slowly squeeze 4 μL of deionized water onto the surface of the chitosan film that was supported on a slowly moving platform. The video was used to immediately capture the image and record the water contact angle. The SCA20 software was used to analyze the captured image and calculate the angle of the droplet contacting the film surface.

### 2.6. Moisture Content

The moisture content of the films was calculated in accordance with GB 5009.3-2016 “Determination of moisture in Food” method [16] by measuring the difference in mass of the sample before and after drying:

(5) $$\mathrm{Moisture}\;\mathrm{content}\;\left(\%\right)=\frac{m_{a}-m_{b}}{m_{a}}\times 100,\;$$

where *m_a_* is the initial sample mass (g) and *m_b_* is the final sample mass (g).

### 2.7. Acetic Acid Content of Chitosan Films

Acetic acid content in chitosan films was determined using high performance liquid chromatography (e2695, Waters, Milford, MA, USA) (HPLC) [17] with a Symmetry C18 (250 mm × 4.6 mm, 5 μm) column. The detection wavelength, column temperature, and flow rate were 210 nm, 25 °C, and 1 mL/min, respectively.

### 2.8. Fourier Transform Infrared (FTIR) Spectroscopy

The FTIR spectra of chitosan films were recorded at room temperature using a Fourier transform infrared spectrometer (Nicolet IS 10, Thermo Electron, Waltham, MA, USA) (FTIR) in attenuated total reflection (ATR) mode with a scanning range from 650 to 4000 cm^−1^ (64 consecutive scans with a 4 cm^−1^ resolution).

### 2.9. Small Angle X-ray Scattering (SAXS)

Chitosan films were cut into strips (2 × 1 cm) and small angle X-ray scattering measurements were performed using a Bruker NanoSTAR SAXS instrument (Bruker AXS, Karlsruhe, Germany). A monochrome Cu-Kα ray with wavelength λ = 0.1542 nm, a current of 600 mA, and a voltage of 50 kV were used in the tests. A one-dimensional function I (q) = (4π/λ) sin θ, as a function of the modulus of the scattering vector, was obtained from the integration of the two-dimensional signal. All of the scattering functions were normalized. A generalized indirect Fourier transform program package was used to process the data and calculate the size of microregion. In addition, the generalized indirect Fourier transform program was used to calculate the pair distance distribution function (PDDF).

### 2.10. X-ray Diffraction (XRD)

XRD diffractograms of chitosan films were obtained using an X-ray diffractometer (D2 Phaser, Bruker AXS, Karlsruhe, Germany). A copper target Cu-Kα (λ = 0.15406 nm) with a power of 1.6 kW (40 kV × 40 mA) was used in the experiment. The X-ray intensity was measured by the NaI crystal counter. The samples were scanned at 5°, with a step of 0.04° and speed at 6°/min, then ended at 60°.

### 2.11. Scanning Electron Microscopy (SEM)

The chitosan film samples were conditioned in an acrylic drying cabinet at 25 ± 1 °C and 52% ± 1% relative humidity for 72 ± 2 h. The samples were fractured in liquid nitrogen and stored in a dryer at 25 °C. Then, the samples were fixed on the sample table and gold was applied under vacuum. The microstructure and surface morphology of chitosan films at different magnifications were observed using a Quanta 200 scanning electron microscope at 1 kV acceleration voltage.

### 2.12. Statistical Analysis

All of the data were recorded as the mean value ± standard deviation. One-way analysis of variance (ANOVA) was performed using the SPSS 24.0 package (IBM, New York, NY, USA). Duncan’s-multiple range test was applied to determine the significant differences between the mean values of each group (*p* < 0.05).

## 3. Results and Discussion

### 3.1. Mechanical Properties

The mechanical properties of edible films are critical for maintaining the structural integrity of food during processing, transportation, and storage [18]. Generally, the tensile strength and elongation at break values are important parameters used to determine the mechanical properties of chitosan films [19].

As shown in Table 1, there are significant differences in the thickness, tensile strength, and elongation at break values of chitosan films prepared under different solution concentrations and neutralization conditions (*p* < 0.05). The concentration of the film-forming solution had little effect on the film thickness. However, the thickness of the chitosan films decreased significantly in value after neutralization, which may be related to the removal of acetic acid. Subsequently, the microstructure of the chitosan films changed when the chitosan films were re-dried and crystallized. This indicated that neutralization had a much greater effect on the thickness of the chitosan films than the concentration of the film-forming solution [20]. For chitosan films before neutralization, the tensile strength increased from 37.6 ± 0.9 MPa to 60.5 ± 3.1 MPa with an increase in the concentration of the film-forming solution. The tensile strengths of the chitosan films before neutralization were much lower than those after neutralization. This may be caused by the presence of acetic acid in the chitosan film, which destroyed the ordered structure of the film, and thus reduced the tensile strength. Neutralization removed the acetic acid in the chitosan films, resulting in an increase in tensile strength. The elongation at break values of the chitosan films decreased significantly after neutralization, which may be related to the change in the molecular chain structure of chitosan and removal of acetic acid.

### 3.2. Wetting Characteristics

Water contact angle (WCA) is an important index used to evaluate the hydrophilicity and wetting characteristics of the film surface. Generally, a WCA greater than 65° indicates hydrophobicity, whereas a value less than 65° indicates hydrophilicity [21]. Packaging films with a higher WCA exhibited stronger surface hydrophobicity and were more suitable for foods with higher moisture contents [22]. As shown in Table 2, the WCA of the chitosan films before neutralization gradually increased in value with an increase in the concentration of the film-forming solution. The WCA values of the chitosan films after neutralization were greater than 90°, which might be due to the removal of acetic acid. For instance, films before neutralization with lower acetic acid contents had greater WAC values. The removal of acetic acid led to strong hydrogen bonds between larger numbers of amino groups on the chitosan molecular chains. These were significantly stronger than the hydrogen bonding between chitosan and water molecules, resulting in chitosan films with greater hydrophobicity.

### 3.3. Water Vapor Permeability

Water vapor permeability (WVP) is an important property for food packaging materials [23,24], since it characterizes the ability of films to inhibit the transfer of moisture between the food and the external environment. The change in WVP of chitosan films was related to the presence of functional groups, such as -NH_2_ and -OH, which were usually the binding sites of water molecules [25].

**Figure 1:**
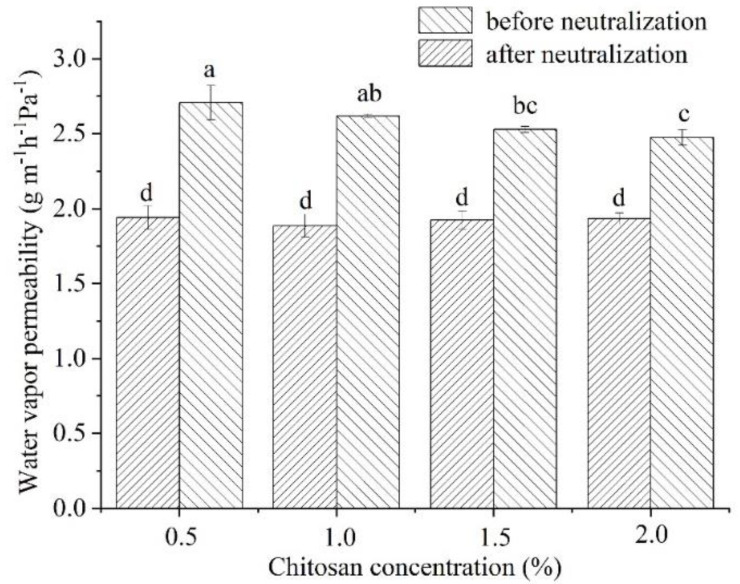
Effects of chitosan concentration on water vapor permeability of chitosan films before and after neutralization. Different letters indicate significant differences (*p* < 0.05).

As shown in Figure 1, the chitosan films before neutralization showed a significant decrease in WVP with an increase in concentration of the film-forming solution (*p* < 0.05). This was consistent with the gradual decrease in acetic acid contents with an increase in chitosan concentrations. This was probably due to the stronger affinity between acetic acid and water in the chitosan films with acetic acid acting as a channel for water vapor in the films. In addition, acetic acid affected the ordered structure of the chitosan film to a certain extent, resulting in a looser structure and an increase in WVP [26].

The WVPs of the chitosan films before neutralization were significantly higher than those after neutralization (*p* < 0.05). Moreover, the concentration of the film-forming solution had no significant effect on the WVP values of the chitosan films after neutralization. Furthermore, this indicated that the acetic acid could affect the ordered structure of chitosan films. In addition, the differences in WVP values between chitosan films before and after neutralization were related to the increase in ordered structures in the neutralized chitosan films after recrystallization and secondary drying.

### 3.4. Moisture Content

**Figure 2:**
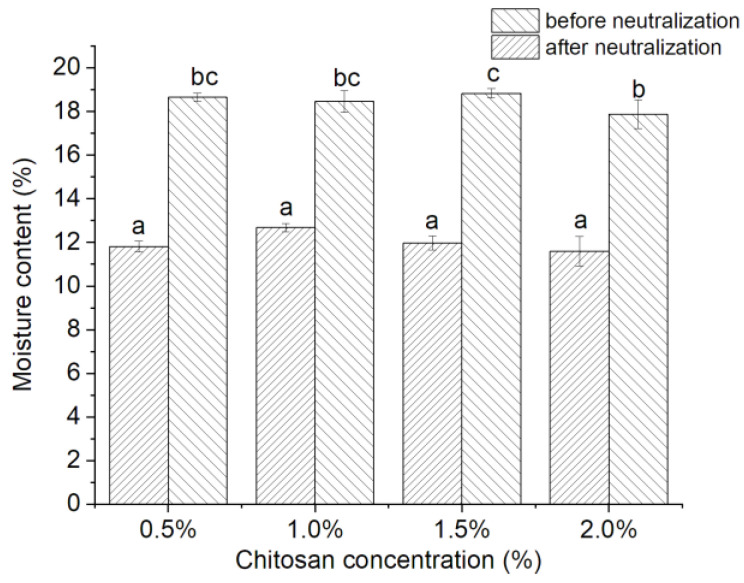
Effects of chitosan concentration on moisture contents of films before and after neutralization. Different letters indicate significant differences (*p* < 0.05).

Chitosan acetate is more hydrophilic than the chitosan molecules and has a greater ability to bind more water, thus increasing the water content in the film. As shown in Figure 2, the moisture contents of the chitosan films decreased from 18~19% before the neutralization treatment to 12~13% after the neutralization treatment. However, the concentration of the film-forming solution had no significant effect on the film moisture content. This may be due to the presence of large amounts of chitosan acetate before neutralization, which was only slightly affected by the concentration of the film-forming solutions.

### 3.5. The Acetic Acid Content

**Figure 3:**
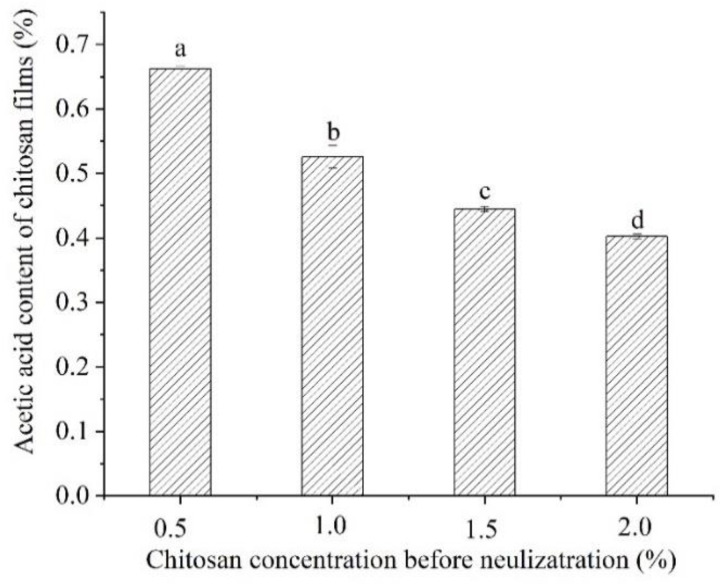
Effects of chitosan concentration on acetic acid contents of chitosan films before neutralization. Different letters indicate significant differences (*p* < 0.05).

The actual contents of acetic acid in the chitosan films are shown in Figure 3. Before neutralization, the acetic acid contents of chitosan films decreased significantly with an increase in the concentration of the film-forming solutions (*p* < 0.05). After drying to form a film, the contents of acetic acid in the chitosan films were greatly reduced compared with the contents in the initial film-forming solutions (1% *w*/*w* in initial film-forming solutions). This might be due to the volatilization of acetic acid during the drying process. Moreover, the presence of acetic acid led to the worsening of the mechanical and barrier properties of chitosan films.

After neutralization, the acetic acid contents in the chitosan films were lower than the detection limit of the high-performance liquid chromatography (HPLC) (10 mg/L), indicating that neutralization could effectively remove the acetic acid in the chitosan films. This was an effective method to reduce the adverse effects of acetic acid in the films. For instance, the influence of acetic acid on the flavor and taste of fresh foods could be reduced, making the chitosan films more effective for preserving the sensory properties of foods.

### 3.6. FTIR Spectroscopy

**Figure 4:**
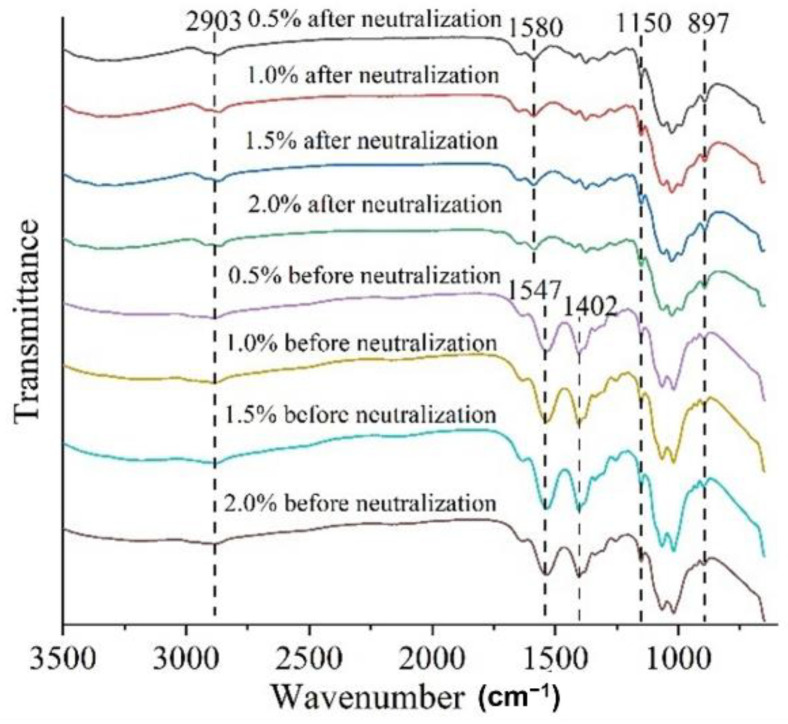
Effects of chitosan concentration on FTIR spectra of chitosan films before and after neutralization.

The infrared spectra of chitosan films before and after neutralization are shown in Figure 4. The peak at 897 cm^−1^ was the characteristic absorption peak of β-glycosidic bond C−O−C [27] and the peak near 1402 cm^−1^ was the in-plane bending vibration peak of the C−H bond in methyl and methylene. The peak near 1547 cm^−1^ was the in-plane bending vibration peak of the N−H bond and the peak at 2903 cm^−1^ was the stretching vibration peak of the saturated C−H bond.

Some FTIR characteristic peaks of the chitosan films changed after neutralization, which might be related to the removal of acetic acid. For instance, the 1402 cm^−1^ peak of the methyl and methylene groups disappeared after neutralization. Moreover, the N−H peak at 1547 cm^−1^ shifted to 1580 cm^−1^. In addition, the intensities of peaks in the chitosan films were reduced after neutralization, which might be due to the disappearance of hydrogen bonds between chitosan and acetic acid. 

### 3.7. Crystal Structure

#### 3.7.1. Small Angle X-ray Scattering (SAXS) Analysis

**Figure 5:**
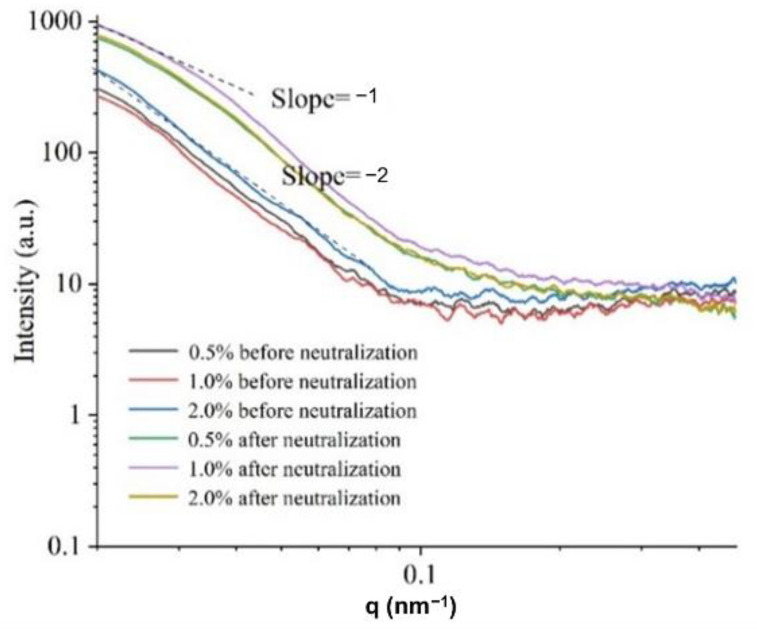
Effects of chitosan concentration on crystals of films before and after neutralization.

The small angle X-ray scattering (SAXS) intensity is based on the differences in electron cloud density at the nanometer scale. SAXS is often used to study the changes in the size and morphology of the ordered structures (crystals) in films [28,29]. As shown in Figure 5, the SAXS spectra of chitosan films had non-linear scattering signals, indicating that nano-scale domain regions existed within each sample. The scattering intensities of the chitosan films before neutralization decayed faster than those after neutralization, suggesting that the films before neutralization had larger nano-scale domains.

The SAXS curves of different film-forming solution concentrations before neutralization shifted slightly up and down, but the shapes of the curves were consistent, indicating that the sizes of the crystals in the films were similar. The slope of the chitosan films before neutralization was −2, indicating that the nano-scale domains within the film may be disc-shaped particles. In comparison, the slope of the chitosan films after neutralization was between −1 and −2, indicating that the nano-scale domains had shapes that were likely between discs and rods [30]. These results indicated that the neutralization treatment was the most important factor that affected the crystal morphology in the chitosan films.

#### 3.7.2. X-ray Diffraction (XRD) Analysis

**Figure 6:**
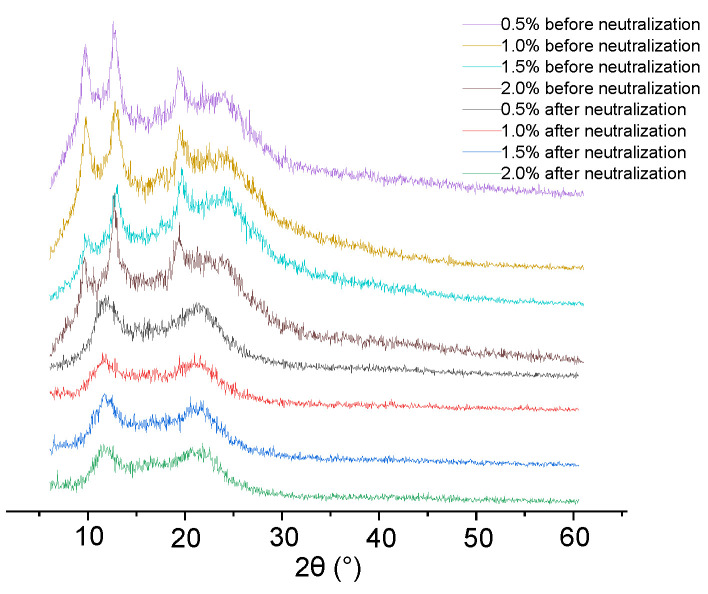
Effects of chitosan concentration on XRD diffractograms of chitosan films before and after neutralization.

XRD was used to study the influence of the film-forming solutions on the ordered crystal structures of chitosan films before and after neutralization. In accordance with Chang et al., chitosan has four crystal forms: Type tendon, type form II, type annealed, and type form 1–2 with chitosan acetate exhibiting characteristic diffraction peaks at 2θ = 12°, 16°, 20°, and 23° [31]. As shown in Figure 6, chitosan films before neutralization had four characteristic peaks at 8°, 12°, 20°, and 23°. After neutralization, the characteristic peaks at 2θ = 12°, 16°, and at 23° disappeared due to the removal of chitosan acetate and acetic acid. Meanwhile, a broad peak appeared at 2θ = 20°, which could be related to the recrystallization of the chitosan films after neutralization. 

### 3.8. SEM Analysis

**Figure 7:**
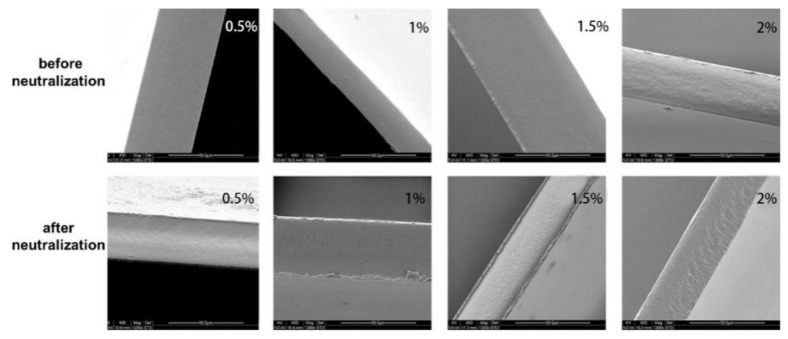
Effects of chitosan concentration on the microstructures of films before and after neutralization.

The chitosan films with different film-forming solution concentrations were pale yellow in appearance before and after neutralization, but they had some differences in their microstructures. Figure 7 shows that the concentrations of the film-forming solutions had little effect on the microstructures of the chitosan films. However, the chitosan films after neutralization were relatively rough. This might be due to the re-drying of the films after neutralization, which induced the formation of more rod-like crystal structures. This increase in ordered structures caused the morphology of the films to be very rough. 

## 4. Conclusions

In this study, we examined the effects of chitosan concentration in the film-forming solutions before and after neutralization. Before the neutralization treatment, an increase in chitosan concentration led to a decrease in water vapor permeability. The acetic acid contents in the chitosan films decreased with an increase in the chitosan concentration. The acetic acid acted as a plasticizer, which affected the cross-linked network structure of the chitosan films to a certain extent, resulting in a looser film structure. After the neutralization treatment, the tensile strength and water contact angles of the films were higher than those obtained before neutralization, which was caused by recrystallization. Moreover, the chitosan concentration had little effect on the properties of neutralized films. These results indicated that neutralization helped in improving the properties of chitosan films.

## Figures and Tables

**Table 1:** Effects of chitosan concentration on tensile strength and elongation at break of chitosan films before and after neutralization.

Sample	Film Thickness (μm)	Tensile Strength (MPa)	Breaking Elongation (EB%)
0.5% (before neutralization)	41.4 ± 0.6 a	37.6 ± 0.9 a	13.1 ± 0.1 a
1.0% (before neutralization)	40.0 ± 0.3 a	43.4 ± 1.5 b	12.1 ± 0.1 a
1.5% (before neutralization)	35.8 ± 0.5 a	51.5 ± 2.6 c	12.8 ± 0.4 a
2.0% (before neutralization)	36.8 ± 0.4 a	60.5 ± 3.1 d	13.3 ± 0.2 a
0.5% (after neutralization)	28.7 ± 2.2 b	115.9 ± 6.5 e	4.7 ± 0.9 b
1.0% (after neutralization)	30.2 ± 5.3 bc	115.2 ± 5.9 e	4.9 ± 0.6 b
1.5% (after neutralization)	25.2 ± 2.0 c	112.3 ± 13.6 e	4.2 ± 1.3 b
2.0% (after neutralization)	25.3 ± 3.9 c	118.6 ± 5.5 e	6.1 ± 2.5 b

Different letters in each column indicate significant differences (*p* < 0.05).

**Table 2:** Effects of chitosan concentration on water contact angles of chitosan films before and after neutralization.

Sample	Water Contact Angle (°)
0.5% (before neutralization)	86.9 ± 0.9 a
1.0% (before neutralization)	85.8 ± 0.7 a
1.5% (before neutralization)	88.3 ± 0.9 b
2.0% (before neutralization)	88.9 ± 0.9 b
0.5% (after neutralization)	93.1 ± 2.1 c
1.0% (after neutralization)	92.0 ± 1.3 c
1.5% (after neutralization)	90.3 ± 1.7 c
2.0% (after neutralization)	91.7 ± 2.3 c

Different letters indicate significant differences (*p* < 0.05).

## Data Availability

Data are contained within the article.
